# Cancer center leadership during large-scale crises: a practical evidence-informed framework with perspectives from the Gulf Cooperation Council

**DOI:** 10.3389/fpubh.2026.1877926

**Published:** 2026-07-17

**Authors:** Naif I. AlJohani

**Affiliations:** Department of Oncology, King Faisal Specialist Hospital and Research Centre, Jeddah, Saudi Arabia

**Keywords:** cancer center leadership, crisis management, emergency preparedness, Gulf Cooperation Council, health policy, health system resilience, oncology, supply chain

## Abstract

Cancer centers are disproportionately vulnerable to large-scale crises—including pandemics, armed conflicts, cyberattacks, and supply chain failures—and while COVID-19 generated valuable crisis guidance for oncology, frameworks tailored to the specific structural vulnerabilities of GCC cancer systems have been limited. The Gulf Cooperation Council region illustrates this gap with immediate, real-time urgency. The ongoing 2026 regional military escalation has reportedly disrupted Strait of Hormuz shipping and Gulf air cargo routes—critical corridors for pharmaceutical cold-chain logistics across all six GCC states—posing risks to essential oncology pharmaceuticals, radioisotopes used in nuclear medicine, and CAR-T manufacturing supply chains. Cancer incidence across the GCC is projected to increase by 60% by 2040, and cancer care remains under-integrated into emergency preparedness frameworks globally. This Perspective proposes a six-pillar crisis leadership framework for cancer center directors and oncology leaders, organized across a four-phase crisis lifecycle (Prepare, Respond, Adapt, Recover), drawing on pandemic-era oncology evidence, international oncology society guidelines, and contextual analysis of the 2026 GCC crisis. The six pillars address the full operational spectrum of cancer center crisis management, from triage and pharmaceutical resilience to workforce wellbeing, digital health, and cross-border coordination. Three specific policy recommendations for GCC health systems are proposed: a formal GCC Oncology Crisis Network with a Regional Real-Time Pharmaceutical Dashboard and distributed reserve framework spanning UAE, Saudi Arabia, and Oman; domestic manufacturing investment for oncology essentials; and a GCC oncology resilience research agenda.

## Highlights

- The framework addresses multiple crisis types—pandemics, cyberattacks, supply chain failures, and armed conflict—with the 2026 regional conflict serving as an illustrative real-time case study in GCC oncology system vulnerability
- A six-pillar crisis leadership framework is proposed for cancer center directors, organized across a four-phase crisis lifecycle (Prepare, Respond, Adapt, Recover)
- GCC cancer centers demonstrated commendable initial resilience during the 2026 conflict, but each country adapted independently—without a pre-agreed regional crisis framework
- Radiological preparedness emerges as a new, lower-probability but high-consequence planning consideration for GCC oncology programs following reported strikes on Iranian nuclear facilities
- A GCC Oncology Crisis Network with a Regional Real-Time Pharmaceutical Dashboard and distributed reserve architecture is proposed as an immediate policy priority

## Introduction

Cancer does not pause during wars, pandemics, or large-scale crises—yet the institutions responsible for its diagnosis and treatment are acutely vulnerable to the same forces that destabilize the societies they serve. The COVID-19 pandemic demonstrated with unprecedented clarity how rapidly a healthcare emergency can disrupt cancer care delivery, with documented treatment delays, missed diagnoses, and excess cancer mortality attributable to disrupted care pathways (1, 2). These harms were not evenly distributed: patients in resource-constrained settings, rural areas, and socially marginal positions suffered disproportionately—a pattern that characterizes every large-scale healthcare crisis. Despite this recognized vulnerability, cancer care remains under-integrated into emergency preparedness frameworks globally (22).

Beyond infectious disease, cancer centers face a broad spectrum of crisis types: armed conflict displacing staff and disrupting supply chains; cyberattacks paralyzing electronic health records and pharmacy systems; and pharmaceutical shortages compromising treatment continuity. The distinct operational demands of each crisis type are discussed within the relevant pillars below.

The Gulf Cooperation Council (GCC) region—comprising Saudi Arabia, the UAE, Qatar, Kuwait, Bahrain, and Oman—presents a particularly instructive context, and one that is no longer hypothetical. Cancer incidence is projected to increase by more than 60% by 2040, from approximately 58,000 to over 94,000 new cases annually (3). Against this trajectory, the escalating US-Iran armed conflict of 2026—encompassing US and Israeli airstrikes on Iranian nuclear and military infrastructure, retaliatory Iranian missile and drone campaigns, and the reported disruption to Strait of Hormuz commercial shipping—has created a live, unfolding healthcare emergency across all six GCC states. The consequences for oncology are immediate and severe. Among GCC states, the UAE experienced substantial reported disruption during the initial conflict phase: infrastructure attacks were most concentrated there, with Dubai and Abu Dhabi international airports—major global pharmaceutical cold-chain cargo transit hubs through which a significant share of GCC pharmaceutical imports are routed, handling temperature-sensitive biologics and oncology monoclonal antibodies requiring 2–8 °C integrity throughout transit—temporarily closed or severely disrupted (4). Reports indicate that air-cargo capacity in the Gulf region fell approximately 79% between February 28 and March 3, 2026, and commercial activity through the Strait of Hormuz declined to an estimated 90% below pre-war levels by mid-March (5). Published GCC oncology experience confirms that cancer services in the UAE, Bahrain, Kuwait, and Saudi Arabia continued without major disruption during the initial conflict phase (6, 7). Reported national adaptations included Emirates Drug Establishment stockpiling, dose-rounding and land-transport rerouting in Bahrain, procurement rerouting through neighboring countries in Kuwait, and Saudi Arabia’s use of redundant Arabian Gulf and Red Sea corridors (7). These adaptations demonstrate what individual systems can achieve—yet each was independently designed, without pre-agreed regional mechanisms for coordinated reserves, cross-border referral, or workforce deployment. National resilience alone is not a substitute for a coordinated regional architecture. Other GCC states face analogous pressures: Kuwait and Bahrain given their maritime proximity to the conflict zone, Qatar through disruptions to its air cargo infrastructure, and Oman, which has assumed a disproportionate role in regional logistics rerouting (5). A further dimension of particular concern in the current conflict: strikes on Iranian nuclear sites at Fordow, Natanz, and Esfahan caused reported localized radiological and chemical contamination confined to the affected facility sites; no off-site radiation increase has been detected, and current risk to GCC populations remains low (8). However, the Bushehr nuclear power plant—operational and as yet untargeted—represents a substantially higher contamination risk if struck (9). While the more immediate and probable threats to GCC oncology services are pharmaceutical supply disruption and workforce instability, the proximity of these strikes warrants that GCC cancer centers incorporate radiological emergency protocols into their crisis plans as a low-probability, high-consequence consideration, given the potential vulnerability of highly immunosuppressed patients during radiological emergencies.

This Perspective proposes an evidence-informed, six-pillar crisis leadership framework for cancer centers, with explicit attention to GCC health system considerations, and offers three specific policy recommendations for regional health authorities. It is intended as a practical resource for cancer center directors, oncology department leaders, and health policymakers operating in any setting where healthcare systems face acute destabilization.

### The impact of crises on cancer care

Patients with cancer are disproportionately vulnerable during crises. Immunosuppression from malignancy and treatment leaves them susceptible to infectious diseases; time-sensitive treatment windows mean that delays in surgery, chemotherapy, or radiotherapy can directly reduce curability. A landmark modeling study found that cancer treatment delays of four or more weeks were associated with significant mortality increases across multiple cancer types (10, 11). The indirect mortality burden from disrupted cancer care pathways during COVID-19 may ultimately rival that of direct viral mortality (1).

Crises also impose severe institutional strain. Revenue from cancer care procedures declines sharply during crises, threatening institutional solvency at precisely the moment when communities most need these services. Workforce vulnerabilities are acutely amplified in the current GCC context. Across the six GCC states, expatriate workers constitute between 40 and 90% of the total population—and a comparable proportion of the oncology workforce. The UAE, where an estimated 88% of residents are non-nationals on employer-sponsored insurance, carries the highest workforce vulnerability: internationally recruited professionals face evacuation advisories and residency uncertainties, and geopolitical instability can terminate both residency and health coverage mid-treatment. These vulnerabilities are not incidental to GCC healthcare planning—they are its defining challenge.

### A six-pillar crisis leadership framework

This article proposes a practical oncology crisis leadership framework—organized across six operational pillars and a four-phase crisis lifecycle—contextualized to the specific structural vulnerabilities of GCC health systems. The framework was developed through a structured narrative synthesis. Peer-reviewed literature was identified via PubMed and Scopus (January 2020–May 2026) using terms for oncology, crisis, pandemic, armed conflict, supply chain, and health-system resilience, prioritizing operational impacts on cancer services; approximately 80–100 publications were screened, of which around 30 directly informed the framework structure. This was supplemented by oncology society guidance (ASCO, EBMT, ASTCT, ASH, ILROG, NCCN) and, given the rapidly evolving conflict, contemporaneous gray-literature sources for real-time logistical observations. Operational domains recurring across these sources—leadership and command, triage, workforce, supply chain, digital health, and inter-institutional coordination—were grouped iteratively until a stable six-pillar structure emerged, which maps onto the WHO health-system building blocks (governance, service delivery, workforce, medical products, information, financing) adapted to oncology, supporting their face validity. The framework spans a four-phase lifecycle—Prepare, Respond, Adapt, Recover—with all pillars functioning simultaneously during active crises. As a narrative synthesis rather than a systematic review, it yields an expert-derived conceptual structure, not an empirically validated instrument.

#### Pillar 1: leadership and governance

Effective crisis leadership in oncology requires decisiveness, psychological safety promotion, and structured delegation (12). The cancer center director should characterize the specific crisis type—each imposes distinct operational demands—and convene a multidisciplinary crisis command team that includes oncology, nursing, pharmacy, administration, and information technology. Communication with staff, referring physicians, partner centers, and patients is never more important than during a crisis. Crisis protocols are best developed, rehearsed through tabletop exercises, and reviewed annually. The director should hold standing representation in the institutional crisis command structure and ensure that institutional crisis plans include radiological emergency protocols—an important new requirement introduced by the reported strikes on Iranian nuclear facilities during the 2026 conflict (8). These protocols should include patient-specific guidance for immunocompromised individuals, given the potential vulnerability of highly immunosuppressed patients during radiological emergencies.

#### Pillar 2: triage and care continuity

Rational triage is the cornerstone of cancer care continuity during resource constraints. The public health stakes are high: modeling studies estimate that a 3-month national lockdown could generate excess cancer deaths equivalent to years of missed diagnoses (1)—collateral mortality that is preventable through structured triage rather than complete service suspension. International consensus across ASCO, NCCN, and ASH supports a structured priority framework: Priority 1 (treat immediately) for life-threatening emergencies; Priority 2 for curative-intent treatments that cannot be deferred beyond 4 weeks; Priority 3 for treatments safely deferrable 4–8 weeks; and Priority 4 for surveillance and non-urgent follow-up. All triage decisions are best made within a multidisciplinary framework, documented with explicit clinical rationale, and applied consistently without discrimination by age, nationality, disability, or socioeconomic status (13). Hypofractionated radiotherapy, oral chemotherapy substitution, and home-based care maximize treatment continuity while minimizing patient contact burden. Surgical deferral is supported by multiple expert groups where clinically feasible, with tumor biology determining deferral duration (14).

**Figure 1:**
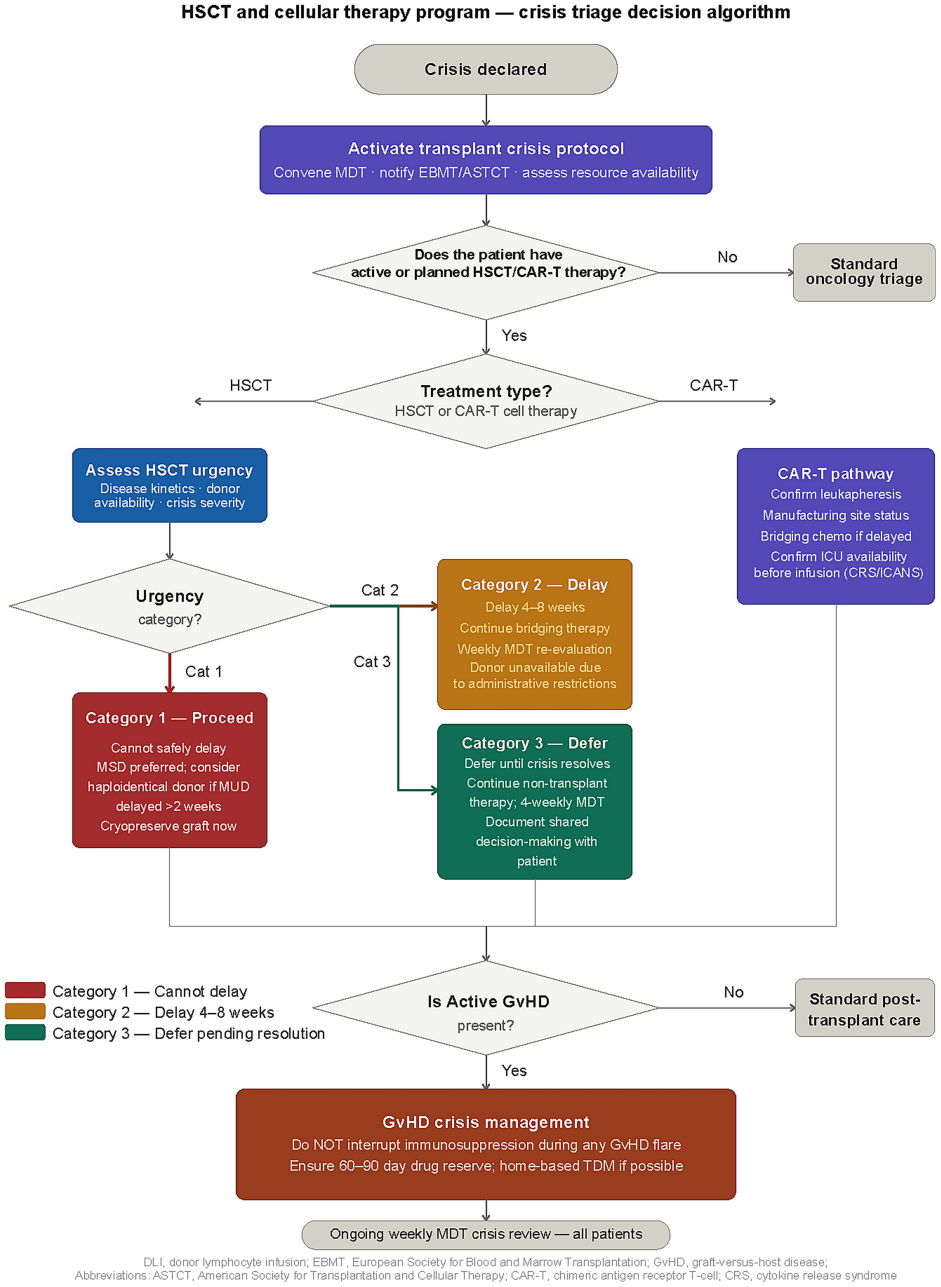
HSCT and cellular therapy program crisis triage decision algorithm. Following crisis declaration and MDT activation, patients with active or planned HSCT or CAR-T therapy are triaged by treatment type. HSCT patients are stratified by urgency into Category 1 (cannot safely delay—proceed with haploidentical pivot if required), Category 2 (delay 4–8 weeks acceptable—continue bridging therapy), or Category 3 (defer until crisis resolution). The CAR-T pathway addresses leukapheresis feasibility, manufacturing site status, bridging chemotherapy, and ICU capacity for CRS/ICANS management. Post-transplant follow-up assessment screens for active GvHD; confirmed GvHD triggers a crisis management protocol with mandatory immunosuppression continuation, 60–90-day drug reserve, and DLI contraindication. Ongoing weekly MDT review applies to all patients. ASTCT, American Society for Transplantation and Cellular Therapy; CAR-T, chimeric antigen receptor T-cell; CRS, cytokine release syndrome; DLI, donor lymphocyte infusion; EBMT, European Society for Blood and Marrow Transplantation; GvHD, graft-versus-host disease; HSCT, hematopoietic stem cell transplantation; ICU, intensive care unit; ICANS, immune effector cell-associated neurotoxicity syndrome; MDT, multidisciplinary team; MSD, matched sibling donor; MUD, matched unrelated donor. This algorithm is a consensus-based conceptual decision aid adapted from existing EBMT and ASTCT crisis-period transplantation guidance; it has not been prospectively validated.

Patients undergoing hematopoietic stem cell transplantation (HSCT) and CAR-T cell therapy require specialized triage protocols, as treatment delays can be irreversible in these populations. A detailed HSCT/CAR-T crisis triage algorithm is presented in Figure 1; this algorithm was adapted from published EBMT and ASTCT crisis-period transplantation guidance and represents a consensus-based decision aid rather than a prospectively validated tool. Table 1 summarizes the six-pillar framework and corresponding GCC-specific operational vulnerabilities.

**Table 1:** Six-pillar crisis leadership framework—summary.

Pillar	Core objective	Key operational actions	GCC-specific vulnerability
1. Leadership & GOVERNANCE	Establish command; characterize crisis type; communicate across all stakeholders	Convene MDT crisis command; rehearse protocols annually; ensure radiological emergency protocols include guidance for immunocompromised patients following reported Iranian nuclear facility strikes (8)	Internationally recruited directors; geopolitical crisis may threaten leadership continuity. New 2026 consideration: radiological preparedness (low-probability, high-consequence)
2. Triage & care continuity	Prioritize care rationally; protect curative-intent patients; maintain HSCT and CAR-T programs	Apply structured HSCT/CAR-T crisis triage (Figure 1); continue MDT tumor boards virtually; document shared decision-making	HSCT and CAR-T programs require uninterrupted international supply chains; uniquely exposed to GCC logistics disruption
3. Staff & wellbeing	Retain workforce; prevent burnout; cross-train for coverage gaps	Create alternating teams; cross-train for essential functions; provide mental health services, flexible scheduling, and family support	UAE: 88% of residents are non-nationals; geopolitical instability risks workforce attrition disproportionate to other regions
4. Supply chain & pharmacy	Maintain essential oncology medication access; prevent critical shortages	Maintain 90-day buffer stock; multi-vendor procurement; adopt standardized inventory reporting format; use verified alternative corridors (Bahrain land, Oman/Muscat, Jeddah/Red Sea)	80% of GCC pharma ($23.7B) routed through GCC airspace/Strait of Hormuz; major air cargo and Hormuz disruptions reported in 2026 (5); CAR-T supply chains at substantial risk
5. Telemedicine & digital health	Maintain care continuity virtually; protect data; sustain clinical trial activity	Scale telemedicine for stable follow-up; community health worker support for digitally underserved patients; paper fallback for cyber events; adaptive protocols and remote monitoring for trials	6.7% of global clinical trials disrupted by 2026 Middle East conflict (19); state-sponsored cyberattacks elevated during active geopolitical conflict
6. Inter-institutional networks	Enable cross-border referral and shared resources—the primary multiplier enabling all other pillars at regional scale	Pre-agree cross-border referral pathways; establish distributed pharmaceutical reserve (UAE, KSA, Oman); implement Regional Real-Time Dashboard under Gulf Health Council governance	No formal GCC Oncology Crisis Network exists; 2026 conflict demonstrated each country adapted independently

CAR-T, chimeric antigen receptor T-cell; GCC, Gulf Cooperation Council; HSCT, hematopoietic stem cell transplantation; IAEA, International Atomic Energy Agency; MDT, multidisciplinary team.

#### Pillar 3: staff and wellbeing

Staff wellbeing is a strategic priority, not an ancillary concern. Fear of infection, family separation, moral distress, and burnout constitute documented occupational hazards during healthcare crises (15). Flexible scheduling, hazard pay, childcare support, and access to mental health services are investments in institutional resilience. In the GCC context, where many oncology professionals are physically separated from their families by international borders, geopolitical instability compounds occupational stress in ways that require explicit institutional acknowledgment. Workforce retention during a crisis is itself a public health intervention.

#### Pillar 4: supply chain and pharmacy

As of early 2026, reported figures indicate more than 270 drugs remain in active shortage in the United States, with 22 chemotherapy agents currently listed and 50% of shortages persisting for two or more years (16). In the GCC, these pre-existing vulnerabilities have been compounded by the 2026 conflict. Because oncology pharmaceuticals for all six states are heavily routed through UAE hubs (Jebel Ali, Dubai, Abu Dhabi), their disruption is a pan-GCC rather than a UAE-specific problem. In the author’s assessment, more geographically distant facilities such as those in Eastern Saudi Arabia may be particularly exposed. Drugmakers have begun diverting shipments through Jeddah, Riyadh, Istanbul, and Oman, but each rerouting adds cost and temperature-excursion risk (5). CAR-T manufacturing supply chains—which depend on uninterrupted air transport of viral vector components from US and European manufacturing sites, with a 4–6-week production window leaving no tolerance for logistics failures—may face substantial disruption risk, with potential implications for program continuity across the region. Gulf-origin petrochemicals, which supply the hydrocarbons used in the manufacture of intravenous bags, vial stoppers, and syringe barrels, have experienced production and export disruptions with downstream implications for injectable oncology preparations. Disruption to cancer medicine supply chains has been independently confirmed by Reuters reporting on Gulf air routes (4) and a Lancet Oncology correspondence specifically addressing cancer medicine supply chain disruption (17). Pharmacy directors are encouraged to maintain 90-day buffer stock where feasible, activate multi-vendor procurement relationships, and convene regular pharmacy-oncology liaison meetings. Verified active alternative corridors include Bahrain land transport, Oman’s Muscat port, and Jeddah’s Red Sea routes for western GCC facilities. To enable the Regional Real-Time Pharmaceutical Dashboard to function effectively, cancer centers should adopt a standardized regional inventory reporting format—specifying drug name, stock level, reorder threshold, and expiry date—ensuring consistent, actionable data across all six states. Reactive panic procurement should be avoided, as it can create secondary shortages.

#### Pillar 5: telemedicine and digital health

Telemedicine is the most significant adaptive tool available during crises. During COVID-19, virtual care utilization increased 135%, with documented benefits to quality of life, psychological outcomes, and geographic access (18). In the GCC, where cancer patients frequently travel significant distances to access tertiary oncology centers, telemedicine for stable follow-up and remote symptom monitoring represents an important structural investment that crisis conditions should catalyze. Equity considerations are important from the outset: older patients and those from lower socioeconomic backgrounds may lack digital literacy or connectivity, requiring language-adapted platforms and community health worker support. For GCC cancer centers with active trial programs, the conflict has created additional research continuity challenges: industry analyses suggest that approximately 6.7% of globally recruiting trials may have been impacted by Middle East disruption, with oncology trials—particularly lung cancer and multiple myeloma—most affected (19). Adaptive protocol planning and remote monitoring infrastructure are now essential components of any GCC oncology crisis plan. Cybersecurity resilience is inseparable from digital health strategy: ransomware attacks on healthcare organizations more than doubled between 2016 and 2021 (20), and state-sponsored cyberattacks during active geopolitical conflicts represent a distinct and escalating threat.

#### Pillar 6: inter-institutional networks

No cancer center can manage a large-scale crisis alone. Pre-established inter-institutional referral frameworks, shared procurement arrangements, and collaborative telemedicine infrastructure substantially reduce the time required to activate coordinated responses (21). In the GCC, where cancer capacity is unevenly distributed across six states, cross-border referral frameworks are essential. The absence of a formal GCC Oncology Crisis Network with pre-agreed referral pathways and shared pharmaceutical emergency reserves represents a critical structural gap—one that the current geopolitical situation makes urgent to address.

### Policy recommendations for GCC health systems

Three specific policy recommendations arise from this framework analysis:

First, health ministries across the GCC should establish a formal GCC Oncology Crisis Network with cross-border referral pathways, shared pharmaceutical emergency reserve standards, and a Regional Real-Time Oncology Pharmaceutical Dashboard providing live visibility into drug stock levels across all six states. The reserve architecture should be distributed—UAE (primary cold-chain hub), Saudi Arabia (dual sea access, multiple airports), and Oman (confirmed active alternative via Muscat port) so that no single country’s disruption collapses regional oncology supply. The Gulf Health Council, as the existing GCC-wide health coordination body, is the appropriate governance home for both the network and the Dashboard, building on established institutional structures rather than creating parallel mechanisms. Given the 2026 conflict’s trajectory toward prolonged, cyclical instability, this network should be designed for sustained operation, not short-term emergency response.

An equity consideration specific to the UAE: with 88% of residents being non-nationals on employer-sponsored insurance, geopolitical disruption can terminate both residency status and health coverage simultaneously. The GCC Network should include a minimum care guarantee ensuring no patient receiving active curative-intent therapy is denied treatment continuity due to insurance disruption arising from the crisis.

Second, domestic or regionally-sourced manufacturing capacity for oncology essentials—including injectable chemotherapy, immunosuppressive agents, and CAR-T viral vector components—should be elevated to a national industrial health policy priority. The current dependence on just-in-time pharmaceutical inventory—often with limited buffer stock under normal conditions (5)—leaves limited margin when multiple supply disruptions converge simultaneously, as the 2026 conflict has illustrated.

Third, GCC-specific crisis management evidence for oncology should be developed prospectively through collaborative research. The current evidence base is dominated by COVID-19 pandemic data from high-income Western settings; its applicability to the GCC context has not been validated. A prospective GCC oncology resilience research agenda—tracking institutional responses, patient outcomes, and workforce impacts during crisis events—would generate actionable, regionally-grounded evidence for future planning.

These three recommendations are conceptual policy proposals, not evidence-validated interventions, and their feasibility would require prospective evaluation. Implementation faces substantial barriers: heterogeneous national drug-registration and regulatory frameworks; the absence of harmonized cross-border licensure for rapid workforce redeployment; the data-sharing, governance, and commercial-confidentiality agreements a Real-Time Dashboard would demand; the financing, cost-sharing, and stock-rotation questions a distributed reserve raises; and the political alignment that sustained coordination ultimately requires. The Gulf Health Council provides an existing foundation that could mitigate some of these, but a phased approach—beginning with voluntary data-sharing and bilateral reserve agreements—is more realistic than a fully integrated network in the near term.

### Limitations

This framework is grounded primarily in COVID-19 pandemic evidence; prospective evidence for other crisis types is sparse, and the framework should be read as broadly applicable crisis-preparedness guidance for which the 2026 regional conflict serves as one illustrative case rather than the sole evidentiary basis. Several limitations warrant emphasis. First, the framework is an expert-derived conceptual structure based on narrative synthesis and the author’s clinical and leadership experience; it has not undergone prospective validation, and the policy recommendations remain conceptual proposals rather than tested interventions. Second, a number of conflict-related observations—including supply-route disruptions, air-cargo reductions, and logistics rerouting—rely on gray literature and contemporaneous media reporting (for example, Reuters, Think Global Health, CSIS, and industry analyses) rather than peer-reviewed sources, reflecting the rapidly evolving nature of the events; these should be interpreted as provisional and subject to revision. Third, GCC-specific oncology crisis data are largely absent from the published literature, and several regional observations draw on institutional experience and contextual analysis. Fourth, the GCC states are heterogeneous in health-system structure, financing, workforce composition, and geographic exposure, so framework elements will require national adaptation rather than uniform application. Finally, single-author perspective articles by nature reflect one leader’s analysis; full implementation of any framework requires interdisciplinary consultation and institutional validation.

## Conclusion

Cancer center leadership during large-scale crises requires systematic preparation, not improvisation under pressure. The six-pillar framework proposed here provides oncology leaders and health policymakers with a practical, evidence-grounded foundation for institutional and systems-level preparedness. At the time of writing, UAE, Bahrain, Kuwait, and Saudi Arabian cancer centers have maintained services through the initial conflict phase—a testament to national investment in healthcare infrastructure and the professionalism of their clinical teams. The same GCC oncology community notes, however, that a prolonged conflict could introduce progressively greater challenges around supply continuity, equipment maintenance, and workforce stability—challenges that regional coordination could help distribute more equitably (7). Of the six pillars, inter-institutional networks (Pillar 6) function as the primary multiplier: without pre-agreed referral pathways, shared reserves, and workforce agreements, preparedness within each pillar remains confined to national borders and cannot scale to meet a crisis that, by definition, crosses them. Investing in a GCC Oncology Crisis Network now, while services are stable, is more practical than attempting to build one under acute pressure, and would extend the resilience already demonstrated through coordination that no single country can establish unilaterally.

## Data Availability

The original contributions presented in the study are included in the article/supplementary material, further inquiries can be directed to the corresponding author.
